# Evaluation of a Seven-Week Web-Based Happiness Training to Improve Psychological Well-Being, Reduce Stress, and Enhance Mindfulness and Flourishing: A Randomized Controlled Occupational Health Study

**DOI:** 10.1155/2013/676953

**Published:** 2013-12-31

**Authors:** T. Feicht, M. Wittmann, G. Jose, A. Mock, E. von Hirschhausen, T. Esch

**Affiliations:** ^1^Division of Integrative Health Promotion, Faculty of Social Work and Health, Coburg University of Applied Sciences, Friedrich-Streib-Str. 2, 96450 Coburg, Germany; ^2^Department Healthy University, Coburg University of Applied Sciences, Friedrich-Streib-Str. 2, 96450 Coburg, Germany; ^3^Institute for Frontier Areas in Psychology and Mental Health, Wilhelmstr. 3a, 79098 Freiburg, Germany; ^4^Division of Social Work, Faculty of Social Work and Health, Coburg University of Applied Sciences, Friedrich-Streib-Str. 2, 96450 Coburg, Germany; ^5^Foundation Humor Hilft Heilen, Dolivostraße 9, 64293 Darmstadt, Germany; ^6^Neuroscience Research Institute, State University of New York, College at Old Westbury, 223 Store Hill Rd, Old Westbury, NY 11568, USA; ^7^Division of General Medicine and Primary Care, Beth Israel Deaconess Medical Center, Harvard Medical School, CO-1309, 2nd Floor, Office 204A, 330 Brookline Avenue, Boston, MA 02215, USA

## Abstract

*Background*. As distress in society increases, including work environments, individual capacities to compete with stress have to be strengthened. *Objective*. We examined the impact of a web-based happiness training on psychological and physiological parameters, by self-report and objective means, in an occupational health setting. *Methods*. Randomized controlled trial with 147 employees. Participants were divided into intervention (happiness training) and control groups (waiting list). The intervention consisted of a seven-week online training. Questionnaires were administered before, after, and four weeks after training. The following scales were included: VAS (happiness and satisfaction), WHO-5 Well-being Index, Stress Warning Signals, Freiburg Mindfulness Inventory, Recovery Experience Questionnaire, and Flourishing Scale. Subgroup samples for saliva cortisol and alpha-amylase determinations were taken, indicating stress, and Attention Network Testing for effects on attention regulation. *Results*. Happiness (*P* = 0.000; *d* = 0.93), satisfaction (*P* = 0.000; *d* = 1.17), and quality of life (*P* = 0.000; *d* = 1.06) improved; perceived stress was reduced (*P* = 0.003; *d* = 0.64); mindfulness (*P* = 0.006; *d* = 0.62), flourishing (*P* = 0.002; *d* = 0.63), and recovery experience (*P* = 0.030; *d* = 0.42) also increased significantly. No significant differences in the Attention Network Tests and saliva results occurred (intergroup), except for one saliva value. *Conclusions*. The web-based training can be a useful tool for stabilizing health/psychological well-being and work/life balance.

## 1. Introduction

### 1.1. Background

Psychology has long been primarily concerned with perception of disorders and negative feelings, but current scientific attention on happiness and the recognition of positive mental states is at an “all-time” high [1, 2]. Reasons for this development are rising levels of psychological pressure and stress at work [3–5], which results in a reduced ability to work due to psychological illnesses. This development is noticeable in Germany. Compared to 1994, the index for days of work missed due to illness increased by 121.1% in 2011 [6]. Since the last decade, researchers increasingly search for answers to the questions of what makes us feel good and how to generate psychological stability and health despite higher work-related demands. Studies showing a link between happiness and vocational success make this a topic of interest for companies and the economy in general as well as for individuals [7]. The investigation of subjective well-being and happiness and the question of whether it is possible to foster positive emotions through training are among the prime topics of happiness studies. Lyubomirsky et al. [8] found that a person's happiness level is determined by 3 factors: a genetic set point for happiness, happiness-relevant circumstances, and happiness-related activities and practices. Furthermore, the authors found that happiness-related activities are the most powerful aspect of increasing and sustaining happiness that is under individual control [7, 8]. Because the brain, principally, never loses its ability to adapt [9], happiness (and, along with it, well-being) is learnable at almost every age; yet activities for pursuing happiness need to be intentional and it takes effort to initiate, carry out, and maintain them [8].

Positive interventions (i.e., positive psychology interventions) help to implement and increase happiness-relevant activities. These are “treatment methods or intentional activities aimed at cultivating positive feelings, positive behaviors, or positive cognitions” [10]. The German happiness training of Dr. Eckart von Hirschhausen is such a positive intervention: a 7-week online training focusing on exercises for achieving a positive psychological state. In addition to an introductory week and a final week, there are 5 weeks with 1 happiness-relevant topic each (e.g., “joy of luck” or “joy of pleasure”). Each week has 3 exercises. The detailed exercises for each week are shown in Table 1. The training is voluntary, free of charge and can be accessed at http://www.glueck-kommt-selten-allein.de.

In this study we aimed to determine the impact of positive interventions in a concrete setting: we examined the effects of online happiness training on occupational health in a company as assessed with questionnaires. We also investigated 2 other aspects. Because the training contains exercises on mindfulness and as studies have shown that mindfulness training enhances the functioning of attention networks [11], we used the Attention Network Test to assess possible effects on attention regulation. To determine if the training reduces stress at an objectively measurable level, we collected saliva samples to measure the stress hormones alpha-amylase and cortisol [12–15].

In a 2011-2012 pilot study, 110 students from Coburg University underwent the happiness training program of Dr. Eckart von Hirschhausen in a randomized controlled trial (unpublished data). The study had some limitations and the hypothesized effects could not be affirmed entirely, but the data clearly showed the effectiveness of the training. Due to this outcome, we then decided to test the training again with a more rigorous design, additional measures, and, particularly, in a different setting (i.e., a more appropriate and presumably a more change/training-sensitive target group), following expected stress and strain levels in employees in the German service sector. We were especially interested in the possible effects of the training on participants experiencing increasing psychological demands at work. Employees of service companies, especially those who provide telephone service, have been among the highest rates of inability to work due to burn-out, depression, or other psychological illnesses in Germany [16]. For this reason, we chose to study employees of an insurance company as our target group, because they are often involved in telephone services and therefore have the potential for improving their work/life balance using an intervention such the happiness training program.

In our paper we followed the CONSORT reporting guidelines as far as possible and useful [17].

### 1.2. Primary Outcome/Hypotheses

The primary objective of this exploratory study is the question whether the happiness training improves individual happiness and satisfaction with life and relieves stress, and, as a consequence thereof, reduces stress-related symptoms in an occupational setting. According to this, our primary outcome was psychosocial well-being, in connection with satisfaction (with life) and stress coping. The assumed effects would be relevant for the field of occupational health and medicine as a whole (e.g., primary care and preventive medicine).

As a secondary objective we investigated how the training influences mindfulness, recovery experience, and flourishing. Furthermore, we tested whether there would be a decrease in objectively measurable stress (measured by saliva samples) or an increase in attentional control (measured by the Attention Network Test).

Taken together, there were 4 aims of this study: *first and as a primary objective,* testing whether the happiness training affected satisfaction and stress; *second,* determining the effects of the training on mindfulness, recovery experience, and flourishing; *third,* investigating effects on objectively measured variables related to stress and attention regulation; and *fourth,* determining if measurable correlations between the analyzed variables exist.

## 2. Methods

### 2.1. Ethics

This study was conducted in 2012 and data were analyzed in 2012-2013. Ethical approval was obtained from the Coburg University Institutional Review Board/Ethics Committee in 2012. In addition, the staff council of the insurance company was officially informed about the study in a premeeting (2012). All participants gave informed consent.

### 2.2. Design

A randomized and controlled longitudinal design (2 groups × 3 times questionnaire/2 subgroups × 2 times saliva and ANT) was used for our study, with 3 different time points (pre = *t*0, post = *t*1, followup (4 weeks later) = *t*2). The intervention group underwent the happiness training partly at work and partly at home (see also Section 2.6.), and the control group did not perform any of the online intervention activities or training and was passive on a waiting-list. After the end of the actual study, however, the control group also did the training (cross-over design); yet, we did not feed the cross-over into the analysis. The reason for the control group formally receiving the training after the actual study period (and nevertheless having this prospective cross-over technically imbedded into the study design right from the start, but no data analysis planned for this) was to guarantee all participants the same experience over the course of the project. This seemed to be appropriate, since it could be expected that being allocated, for example, to the intervention group could produce somewhat “visible” behavior changes, and so it rendered important to not produce a “loser” group by random allocation to the control group. In fact, all participants could be sure that they would, finally, get the same experience (happiness training), if they wanted.

The questionnaires were completed at *t*0, *t*1, and *t*2. The saliva samples and the ANT were only taken at *t*0 and *t*1.

### 2.3. Participants/Recruitment

**Figure 1:**
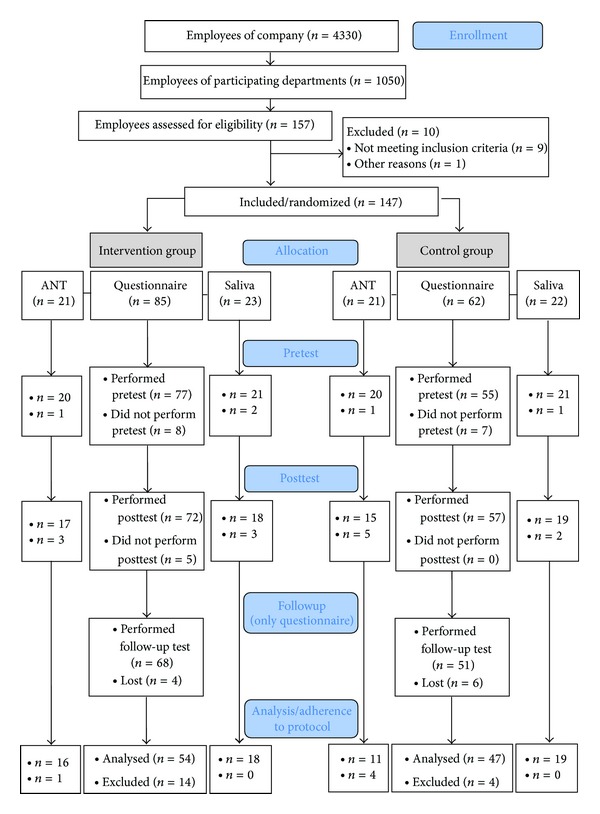
Study and participant flowchart.

The sample consisted of 147 German-speaking, employed, adult volunteers from 2 departments of a local insurance company with a total of 4330 employees. The 2 participating departments were chosen by the company and we had no influence on this decision. In total about 1050 employees of the two departments were possible participants due to the same hierarchy level. All of them had been invited for voluntary participation. 15% of them wanted to participate, were proved to be eligible, and were thus included for randomization (Figure 1). The employees of the 2 participating departments were addressed and informed about the study by their group leader. Inclusion criteria were (1) regular access to the Internet at home (as they performed the training partly at home and partly at work), (2) no vacation of longer than 1 week during the survey period, (3) no prior knowledge of or experience with the online happiness training program. Every participant gave informed consent by formally subscribing to the training and they all participated in an introductory class in which they had the opportunity to individually ask the project team questions.

### 2.4. Randomization/Allocation

Concerning the classification of the participants into intervention (IG) and control group (CG), we also considered local and structural conditions of the company. To avoid direct communication between IG and CG (e.g., when IG participants worked on instructions from the web-based training program at their desks, potentially sitting next to each other) we differentiated between participating sites. We had 2 different departments signing up. One department had open (cubicle) offices, the other one consisted of smaller offices with 2 to 6 employees per room. To prevent a direct influence between IG and CG, we stratified for groups, so that participants in one office were in the same group and not in “competing” groups. As not all employees of each department decided to participate in the study, there were always some nonparticipants in the offices. Hence, randomization took place at an individual level, but via formation of strata. Careful stratification procedures were applied in the process and prior to it, due to given practical/structural constraints and statistical considerations. Yet, we established “virtual” study population compartments that, following department structures in the company, were derived from department affiliation of participants. An additional goal of the illustrated processes was to carefully avoid hierarchic structures or uneven distribution of hierarchy positions and functions between intervention and control groups.

To summarize related randomization procedures, we performed, within the stratified compartments established by our statisticians, individual-level randomization, that is, allocation either towards IG or CG. Moreover, we made sure that in case participants worked in smaller offices (size of, e.g., 2 people) and share direct office space that both participants were allocated to the same group. In this regard, our study compartments (strata) were “physical.” The reason for these somewhat complex measures was to keep spill-over effects from IG to CG reasonably small.

In addition to the general study group allocation we had 2 subsamples: (1) from which saliva was collected and (2) who performed the Attention Network Test (ANT). The subgroups were composed of participants from only one department. We split all participants of this department and randomly assigned them either to ANT or saliva testing; therefore, both subgroups were nonoverlapping.

Randomization procedures were performed by drawing pieces of paper (in lots) from a bag (an untransparent cloth bag). All lots had the same look and were put into the bag and mixed thoroughly by one of the authors. The bag was provided by the author and a noninvolved person was drawing the lots (blinded).

The 147 participants were split into an intervention group with 85 participants and a control group with 62 participants. The difference in size between the groups is due to the different size of the open space offices in the company. See the flowchart for participation and drop-outs (Figure 1).

### 2.5. Instruments

Happiness and well-being are predominantly subjective measures, which need to be defined from the subjective perspective of a person. For this reason, we used an online questionnaire consisting of different subquestionnaires to collect individuals' self-reports for our primary/secondary objectives. For our primary objective we used the following.

At first, we used a Visual Analog Scale (VAS), which asked the question “How happy are you right now?” and another asking “How satisfied are you right now?” on a 6-point rating scale with a sad face on the left and a happy face on the right.

The WHO-Five Well-being Index (WHO-5) was derived from a larger scale originally developed for a WHO project studying diabetes patients [18]. WHO-5 is a generally accepted quality of life/well-being screening instrument consisting of 5 questions developed by the World Health Organization (Psychiatric Research Unit, WHO Collaborating Center for Mental Health, Frederiksborg, Denmark (German version from 1998) [19–22]. It covers positive mood (good spirits, relaxation), vitality (being active and waking up fresh and rested), and general interest (being interested in things) on a 6-point Likert scale from 0 (not present) to 5 (constantly present). In our study it was used to evaluate the general quality of life and changes during the treatment. Example items are “I woke up feeling fresh and rested” or “My daily life has been filled with things that interest me.”

The Stress Warning Signals Scale (SWS) is a list of 61 stress warning symptoms divided into muscular reactions, vegetative-endocrinological reactions, and cognitive, emotional, and behavioral reactions. Using the SWS, data on the subjective stress experience of the participant was collected. The rating follows a 10-point system (0 = does not stress me at all, 10 = stresses me a lot). The scale was developed and validated by Esch et al. [23, 24]. Example items for vegetative-endocrinological symptoms are “rapid heartbeat,” “chest tightness,” or “excessive sweating.”

For testing the impact and determining factors of the training (secondary objective), we used the following additional questionnaires:

The Freiburg Mindfulness Inventory (FMI) [25] is a 30-item scale measuring mindfulness with a 4-point Likert scale (1 = rarely, 4 = almost always). We used the 14-item short version. The FMI is a useful, valid, and reliable questionnaire for measuring mindfulness. It is most suitable for use in generalized contexts in which knowledge of the Buddhist concept of mindfulness cannot be expected [25]. The 14 items cover all formal aspects of mindfulness. Example items are “I am open to the experience of the present moment” and “I am friendly to myself/do not criticize myself when things go wrong.”

The Recovery Experience Questionnaire (RECQ) [26] is a valid and reliable questionnaire for use in assessing an individual's unwinding (i.e., relaxation or “wind-down”) and recuperation processes. It measures the elements of “psychological detachment from work,” “relaxation,” “mastery,” and “control” on a 16-item scale with a 5-point Likert scale (1 = not at all, 5 = very much). Example items are “In leisure time I forget about work” and “In leisure time I decide my own schedule.”

The Flourishing Scale (FS) [27] is an 8-item measure of psychosocial well-being and personal growth and development (i.e., flourishing). A pilot study showed internal consistency and a moderately high reliability and validity [27]. We used a German version of this questionnaire (FS-D) translated by Esch et al. [23, 24]. Example items are “I lead a purposeful and meaningful life” and “I am engaged and interested in my daily activities.”

In addition to the subjective reports of our participants, we wanted to know if there were any objective physiological or behavioral changes that could be detected. Therefore we analyzed the effects of the happiness training on 2 objective parameters. Two subsamples were built of participants from one department. The subgroup testing was only performed at pre- and posttest (no followup).

From one subsample (*N* = 45), we collected saliva at 3 different times a day (directly after awakening, 30 minutes later, and at 8 pm) for analyzing saliva concentrations of cortisol and alpha-amylase, which are indicators for the activity of the 2 stress axes in the body [13–15, 28]. It has long been known that cortisol (i.e., elevated plasma levels that are positively correlated with elevated saliva concentrations as indicators, e.g., for the hypothalamic-pituitary-adrenal stress axis) can be an indicator of stress activity. Only recently, amylase received special attention in this regard, since its saliva levels seem to correlate with sympathetic-adrenal medullary system activation as an indicator for the sympathetic arm of the stress response [13–15, 28–31], including positive correlations with norepinephrine/(nor) adrenaline levels. However, since salivary alpha-amylase is not only influenced by (sympathetic) stress, with higher levels suggesting sympathetic activation, but also by salivary flow rate (which is more associated with parasympathetic activity), this parameter is still under scrutiny. In any case, stress seems to modulate alpha-amylase levels, as, for example, higher chronic stress seems to correlate with more deteriorated cortisol and amylase regulation [15, 28, 30, 31], or even attenuated or flattened (but prolonged) response curves. Moreover, acute stress (also, acute stress responsiveness) in general correlates with higher hormone levels. While still being speculative in some areas of interpretation, we nonetheless included these salivary stress parameters in our study to learn more about a possible influence of a potentially stress-reducing web-based happiness training on the stress physiology. Therefore, participants of the saliva subgroup received 6 salivettes (3 × pretest, 3 × posttest) for collecting saliva, as well as detailed information on how to take the samples, before the pretest.

The second objective instrument was the Attention Network Test (ANT; [32]), which was conducted within the other subsample (*N* = 42). Within a 30-minute testing session, the test assesses the functioning of 3 attention networks in anatomical and functional terms: alerting, orienting, and executive control [32, 33]. Reaction time measures for differently cued and uncued stimulus conditions are used to quantify the processing efficiency of these 3 networks. Studies have shown that mindfulness training enhances the functioning of these attention subsystems [11]. Because the happiness training contains exercises on mindfulness, we used this test to examine possible effects.

### 2.6. Intervention

During the survey period, the participants received an email reminder of the next survey point and they had the opportunity to contact the researchers via email for technical support. All participants took part voluntarily and neither gave nor received money to participate. There was an introductory event for giving necessary information; at the end of the study (6 months after formal termination), participants received a thank-you present and were oriented about preliminary results.

One day after the introductory event, the pretest was conducted: all participants filled out the online-questionnaires and the 2 subsamples gave the saliva or performed the ANT (*t*0). Then, the happiness training started and ran for 7 weeks for the intervention group. During this time, the IG received an email every week at work, explaining the current topic and the 3 exercises (time required: approximately 10–15 minutes, once a week). The study of the documents pertaining to the current topic was done during working hours and the performance and documentation of the exercises took place at home during free time. The control group, which was on a waiting list, was a passive group and did not perform any of the online intervention activities or training. The posttest (*t*1) was completed at the end of the training period, which was 7 weeks after *t*0. Again, the online questionnaire, the saliva samples, and the ANT were performed. The last survey was conducted 4 weeks after the end of the training (followup; *t*2). At this point only the online questionnaire was completed (no saliva and ANT test).

Filling out the questionnaire and doing the ANT was done during working hours but the saliva samples were collected at home because the times when they needed to be taken were not during working hours.

### 2.7. Data Analysis

In a first step, the group samples were analyzed according to drop-outs (Figure 1). We analyzed the data following adherence-to-protocol: those participants who completed all tests were evaluated. This means that for the analyses of the questionnaire, all participants who filled out the questions at all 3 time points (*t*0, *t*1, and *t*2) were included. There was a total drop-out rate (from randomization to analyses) of 31.3%. Of these 46 drop-outs (IG: 31, CG: 15 drop-outs) 15 participants decided not to enter the study (reasons included illness, participants did not show up, or decided to extend vacation during the survey) and did not even perform the first test. During the training (*t*0-*t*1) there were 5 drop-outs (paradoxically there were 2 drop-outs more at *t*0 than at *t*1 in the CG. Reasons for this could be that participants performed the questionnaire at *t*0 but missed to press the “send” button at the end, so that the data was not transferred). Another 10 drop-outs occurred during followup. Due to adherence-to-protocol procedure there were 18 drop-outs.

With the subgroups,all subjects who participated at *t*0 and *t*1 were included in the analysis. Saliva samples had a drop-out rate of 17.8% (*N* = 37; IG: 18/CG: 19) and *ANT* had a drop-out rate of 35.7% (*N* = 27; IG: 16/CG: 11).

We compared between-group values (IG versus CG) for *t*0, *t*1, and *t*2, as well as within-group values for IG at *t*0-*t*1, *t*1-*t*2, and *t*0–*t*2. Because some of the data was not normally distributed, we used nonparametric tests for the analyses. The between-group effects were computed with the Mann-Whitney *U* test and the within-group effects were computed with the Wilcoxon test. With the nonparametric statistics the rank order values are compared and statistical measures of central tendency such as mean or median are used for descriptive statistics. We computed effect sizes after Cohen's *d* because we had a rather small sample. An effect size of 0.2 describes a small effect, 0.5 describes a medium effect, and 0.8 describes a large effect.

For the questionnaires, the sum of scores of each single subquestionnaire was computed and compared.

Saliva samples were analyzed at the laboratory of Professor Dr. Clemens Kirschbaum at the Technical University of Dresden. We collected data at time of awakening (*t*_*s*_0), 30 minutes later (*t*_*s*_1), and at 8 pm (*t*_*s*_2) and then calculated the awakening response (*t*_*s*_1 minus *t*_*s*_0), the morning activity (the mean of *t*_*s*_0 + *t*_*s*_1), the evening activity (*t*_*s*_2), and the circadian rhythm amplitude (morning minus evening activity). We chose this calculation of the raw salivary data to be able to associate the measurements with other parts of the questionnaire.

The ANT results were analyzed according to its standard procedures. We computed the parameters of alertness, orienting, control, overall reaction time, and accuracy.

## 3. Results

### 3.1. Questionnaire

Descriptive statistics in the form of means and standard deviations for both groups at all times and differences in the outcome measures for IG versus CG are presented in Table 3. Within-group effects of the IG are presented in Table 4.

Table 3 shows that mean levels of happiness, satisfaction, and quality of life increased during the training for the IG, and only slightly decreased during the follow-up period. The same applies to the Stress Warning Signals—the means of the stress symptoms decreased numerically over the course in sum and in all subcategories in the intervention group, whereas it increased in the control group. Also, the factors mindfulness, recovery, and flourishing increased numerically from *t*0 to *t*1 in the IG, and their means at *t*2 were always superior or similar to *t*1. In the control group, all those subjective parameters decreased or remained the same. For all parameters the improvement in the IG from *t*0 was still visible at *t*2.

#### 3.1.1. Between-Group Analyses

Table 3 also shows the results of the between-group analyses. The effects were analyzed with the nonparametric Mann-Whitney *U* test (as data was not normally distributed) and revealed significant effects over the test period. The Mann-Whitney *U* test at *t*0 shows that at the beginning the 2 groups did not differ. At *t*1, effects were disclosed: satisfaction and quality of life differed significantly, with effect sizes of >1 and *P* < 0.0001. Additionally, happiness (*U* = 603, *P* = 0.000, *d* = 0.93) and, among the stress warnings signals, particularly emotional stress (*U* = 757, *P* = 0.000, *d* = 0.69) improved significantly. The training also showed a strong positive effect on mindfulness (*U* = 866, *P* = 0.006, *d* = 0.62), recovery experience (*U* = 824.5, *P* = 0.002, *d* = 0.63), and flourishing (*U* = 951, *P* = 0.03, *d* = 0.42). Even at *t*2, most variables (happiness, satisfaction, quality of life, Stress Warning Signals (except for muscular and mindfulness)) showed significant differences, with large effect sizes. Recovery experience, flourishing, and muscular stress did not show any significant differences between groups at time point *t*2.

#### 3.1.2. Within-Group Analyses

We used the nonparametric Wilcoxon test for calculating within-group differences. Table 4 gives an overview of results. The table shows good medium effects between *t*0 and *t*1, especially for happiness (*Z* = − 3.63, *P* = 0.000, and *d* = 0.72) and satisfaction (*Z* = − 3.64, *P* = 0.000, and *d* = 0.72), but also for quality of life (*Z* = − 2.88, *P* = 0.004, and *d* = 0.51). A similar outcome shows the sum of the Stress Warning Signals (SWS), with *Z* = − 2.51, *P* = 0.012, and *d* = 0.42. In the categories of SWS, especially the emotional and behavioral symptoms decreased (*Z* = − 2.95/−2.35, *P* = 0.003/0.019, and *d* = 0.46/0.45). The variables of flourishing (*Z* = − 3.13, *P* = 0.002), mindfulness (*Z* = − 2.57, *P* = 0.010), and recovery experience (*Z* = − 2.35, *P* = 0.019) showed a significant difference, with medium effect sizes of *d* ≥ 0.5.

### 3.2. Subgroups

#### 3.2.1. Saliva

Saliva analyses for cortisol did not show any significant differences between groups. The analyses for alpha-amylase showed 2 effects—the awakening response between groups at *t*1 was marginally significantly different (IG *t*0: 7.18, *t*1: −12.41; CG *t*0: 4.97, *t*1: 17.77; *U* = 112; *P* = 0.073; *d* = 0.6) and the morning activity within the IG between *t*0 and *t*1 was significantly different, with *P* = 0.002 (*Z* = − 3.11; *d* = 0.83; IG *t*0: 35.67; *t*1: 59.95).

For more results see Table 5.

#### 3.2.2. ANT

The subsample of participants that performed the Attention Network test had a high drop-out rate (37.5%). At the end, only 16 subjects of the IG and 11 subjects of the CG could be analyzed. Due to technical problems, some of the data had to be excluded. Furthermore, the mean age differed significantly between IG (*M* = 39.6 years) and CG (*M* = 29.6 years). Thus the IG was on average 10 years older than the CG in this subsample.

We found significant changes from *t*0 to *t*1 within IG (Wilcoxon *Z* = − 2.4, *P* = 0.016) and CG (*Z* = − 2.8, *P* = 0.04) for executive control and the accuracy of responses (IG: *Z* = 2.6, *P* < 0.008; CG: *Z* = 2.7, *P* < 0.007), indicating a general learning effect, but no group differences at *t*0 and *t*1. The overall reaction time between *t*0 and *t*1 was significantly lower only for the CG (*Z* = 2.9, *P* = 0.003), and the orienting effect was smaller at *t*1 than at *t*0 only for the IG (*Z* = 2.8, *P* = 0.006). On closer examination of the descriptive statistics, however, no meaningful difference between IG and CG for the orienting effect was detectable.  Table 6 gives an overview of results of the ANT.

### 3.3. Correlations

We conducted bivariate Spearman correlations of the intervention group differences between *t*1 and *t*0 and found some meaningful significant associations. The most important ones are listed in Table 7. We found the most significant correlations between satisfaction/happiness and quality of life (*r* = 0.400/*r* = 0.375)/mindfulness (*r* = 0.445/*r* = 0.301). Additionally, the Stress Warning Signals (especially the emotional signals) correlated with quality of life (*r* = −0.446), and happiness and satisfaction correlated with SWS (*r* = −0.517/*r* = −0.484). The strongest correlation existed between flourishing and mindfulness (*r* = 0.600), and between satisfaction and the morning activity of alpha-amylase (*r* = 0.850). The variables happiness and satisfaction also correlated with each other (*r* = 0.575).

## 4. Discussion

The study outcomes emphasize the health promoting potentials of positive interventions—in this case, with reference to occupational health and to the specific web-based happiness training of Dr. Eckart von Hirschhausen. All surveyed variables of health and/or individual well-being in the online questionnaire showed significant positive effects for the intervention group (IG) between *t*1 and *t*0. This effect between *t*1 and *t*0 was not detectable in the control group (CG). Moreover, at *t*1, the IG showed positive differences in all variables of primary concern as compared to the CG with remarkable effect sizes (e.g., *d* > 1 for satisfaction and quality of life, with *d*> or = 0.8 being a large effect).

The intervention study was conducted during the most stressful period of the year in the company due to a very high workload. This means that the stress level at work was constantly high during our investigation, especially between *t*0 and *t*1. For this reason, we decided to calculate not only between-group effects, but also within-group effects for the IG. Even failing to find a decline of health/well-being in the IG could have been interpreted as a success. Most of the means of the CG in the questionnaire show a decline from *t*0 to *t*1, which indicates the influence of the heavy workload phase. But despite the high “external” stress levels (i.e., circumstantial strain), an improvement in IG was found, which was most visible in between-group analyses. As primary outcomes the subjective feeling of happiness and satisfaction increased significantly, with large effect sizes. It could be argued that the Visual Analog Scale might not be the most appropriate tool, but as the gold standard of research on happiness states, it covers an important field of the participants' self-report [34, 35]. More solid evidence is provided by the WHO-5 Questionnaire; these 5 questions are psychometrically valid to assess well-being state and/or to screen depressed mood [19–21, 36, 37]. Our significant changes within the WHO-5 can be interpreted as a medically relevant outcome, which could also be relevant for primary care. This result indicates that the online training potentially makes the participants psychologically healthier on 2 levels: subjective and “medical” (with reference to depressive states).

Because mind and body are inseparable, it is not surprising that the positive effects on subjective and mood-related experience, as measured in our study, also had an impact on (or were correlated with) awareness of physiological levels. Being psychologically healthier and happier is associated with the awareness of physiological changes (e.g., as seen in the decrease of stress warning symptoms). Especially emotional reactions seem to become more stable after the happiness training. This is hardly surprising, because these specific emotions are closely connected to overall happiness and well-being. Furthermore, the cognitive and vegetative-endocrinological reactions especially improved.

In the *ANT* we could not find any significant effect of training, which was probably due to the high drop-out rate and the small number of analyzable participants. The big difference in age (the IG was on average 10 years older than the CG) might have influenced the results as well, although other studies have shown that there is no noteworthy difference in attentional capacities between subjects 30 and 40 years of age [38]. The ANT is one of the most valid and reliable instruments for testing attention regulation and has been used in other studies successfully testing the positive effects of mindfulness on attention [11, 39]. In those studies the training of mindfulness was much more intensive. Our training used exercises that fall within the scope of mindfulness, but the training itself might be insufficiently mindfulness-based and too short to achieve significant effects with the ANT. Therefore, training might need to be more mindfulness-based, more intense, and conducted over a longer period of time to achieve ANT effects.

Regarding the *saliva* analyses, we tried to measure possible objective effects of the 2 stress axes in the body. The samples were taken at *t*0 and *t*1, each time immediately at awakening, 30 minutes after awakening, and at 8 pm. Due to the study design and the fact that the participants took the saliva samples at home without supervision, it was not possible to precisely control the exact time when the samples were taken; therefore, there might have been some unknown variations in time of saliva collection. We measured the concentration of cortisol as a parameter for the hypothalamic-pituitary-adrenocortical axis (HPA) [14], and alpha-amylase as an indirect parameter of (nor) adrenalin, which is a parameter of the sympathetic-adrenal medullary (SAM) axis [13]; see Section 2.5. The SAM is responsible for the short-term stress reaction, the so-called fight-or-flight response [5, 12]. This means that adrenalin increases faster than cortisol in a stress situation because it is a more direct parameter of short-term stress. We found some significant effects, for example, with the amylase morning activity in the intervention group (*P* = 0.002, *d* = 0.83). The morning activity is an established value and comprises of the sum of the “awakening value” and the “plus thirty value.” The value of morning activity increased in IG from 35.67 U/mL at *t*0 to 59.95 U/mL at *t*1, possibly indicating a “healthy”—that is, physiological, well-functioning—regulation (see above), however, with lesser “alertness” (in absolute degrees) than in CG. This effect, compared with the overall tendency of amylase values in IG to show less responsiveness in IG, for example, decreased awakening response, in combination with measured outcomes of lower vegetative-endocrinological stress symptoms, could be interpreted, but with caution, as a sign that the participants of IG possibly started their day being less alert or “stressed.” However, this is a more physiological interpretation due to the knowledge of the 2 stress axes. The evening effect did not show any significant difference or correlation. The morning effect could thus, quite possibly, be influenced—or “produced”—by better recovery (see next passage) in the evening/during the night and could yet be lost during the day. Since these results and their theoretical association with a still vague knowledge in the field of amylase-cortisol-stress pathophysiology are rather speculative, they can only be seen and carefully be interpreted as “piloting insights,” certainly warranting further and more thorough investigation.

A crucial question is *which determinants* of the training led to the measured effects (secondary objective). Possible answers may be offered by results from the other questionnaires: the study results show that the participants who were trained (IG) felt an increase of mindfulness between *t*0 and *t*1, more than the CG. Furthermore, an increase of the recovery experience can be seen between groups at *t*1. It may have been easier for the IG to “disconnect” or detach from work, relax, and assume control in leisure time to meet new challenges while they underwent the training. Perhaps they fell back into old patterns after the end of the training, which could be the reason why there was no comparable effect at *t*2. Likewise, the Flourishing Scale showed significant differences within the IG between *t*0 and *t*1 and between *t*0 and *t*2, as well as between groups at *t*1. Maybe those exercises act as a “door opener” for personal development and self-care [9] and possibly the instructions were helpful or were the basis for that progress.

The dependent variables assessing psychological well-being correlate with each other as effects of the happiness training. Happiness and satisfaction were especially strongly correlated with quality of life and mindfulness. It seems as if the IG “learned to see and appreciate” the little things of everyday life, which draws attention to more positive experiences, which again could make individuals more happy and thankful; however, this is rather speculative. The subjectively positive experience also spread to physiological signs: quality of life, happiness, and satisfaction were strongly correlated with the Stress Warning Signals—the happier the participants were, the fewer stress warning symptoms they described. The strong correlation between mindfulness and flourishing may describe a higher “spiritual level”; again, this is a rather speculative assumption. By undergoing the happiness training, the participants perhaps got in touch with themselves and their surrounding and may have realized that they can influence and control, at least to some extent, their environment. Another strong correlation exists between the morning levels of alpha-amylase and satisfaction, so there may be a connection between getting up, feeling less arousal, and feeling satisfied.

Some participants reported a strong subjective and positive reaction upon simply answering the questionnaires. Taking questions obviously made individuals reflect on themselves and their lives and some may have realized, just by filling out the questionnaires, that they were not happy at all. This may have resulted, in some cases, in changes in their lives or life-styles (in a “positive intervention direction”). The fact that the participants, including those in the control group, effectively changed some aspects of their daily life experience, as occasionally reported by participants and obviously due to the “encounter” with our study battery could confirm that the measured effects and effect sizes (IG versus CG) are “real”.

It is possible that the training also initiated a “cultural change,” possibly involving an improved work “climate” as incidentally reported by participants, through a conceivable domino effect caused by the intervention group and common hubs at the company (e.g., meeting each other at the cafeteria). This would, once again, confirm the statistically measured differences between the IG and CG.

As the drop-out rate was quite high it might be suggested that an intention-to-treat analysis could have been more suitable than our chosen analysis procedure (adherence-to-protocol). The imputing of missing values (filling in) is sometimes seen as a possibility to replace missing values. However, although this imputing technique of the “last observation carried forward” is used, this approach has serious drawbacks such as those participants who have been replaced with their own data contribute to results through untestable assumptions [40]. By using adherence-to-protocol analyses we tried to prevent the use of untestable assumptions.

In our study we did not investigate possible confounders like age or sex. This was a “specified” setting study, which is for the first time testing the hypotheses/outcome of efficacy of a web-based happiness training in the work environment, therefore applying rigorous RCT methodology. Accordingly, the study cannot be seen or interpreted as an inductive, quasi-experimental design, much more as a first pilot study in the area requested. However, due to practical and structural constraints produced by the chosen setting, and the actual company at hand (yet, we still consider the chosen company a “best possible choice”), we had to opt for complex randomization and stratification procedures, as described. Here, our first goal was to achieve same-hierarchy, same-stress level and reporting categories (strata); therefore we did not adjust or stratify for age and sex. This also met the company's policies for not discerning results by gender. We could have adjusted and controlled for age, but we did not want to produce “overstatistics,” that is, providing too many different analysis procedures.

### 4.1. Limitations

Some limitations of our study must be mentioned. Our findings are based on a sample that participated voluntarily and consisted largely of females (see Table 2). The drop-out rates were comparably high; however, both groups (IG and CG) where affected likewise. The drop-outs resulted in especially low subsamples for the saliva and attention subgroups.

Moreover, because there were no timekeepers attached to the saliva boxes and participants took saliva samples at home without supervision, we cannot guarantee that the saliva samples were always collected at the correct time.

Since we used a waitlist control group design and not an active control, measured effects between the IG and CG could be explained partly by expectation effects.

Furthermore we did not perform formal sample size estimation. Sample size considerations would have, in this particular case, contravened actual randomization procedures, and other on-site needs, since the department and division structures of the company, as well as the actual outlet of diverse facilities, were the main determinant for feasibility in this regard. However, we tried to acquire the biggest number of participants achievable. Besides that, comparable studies in the same area have not been undertaken so far with regard to web-based and occupational health-based, happiness-related “positive interventions.” As a consequence, our study could be interpreted with caution as a pilot study that sparks interest in further research.

Another limitation of our study is no adjustment for potential confounders, for example, age or sex. Further details are mentioned in the discussion.

These limitations could have influenced the results.

## 5. Conclusions

The online happiness training led to higher scores in the self-report measures of happiness and life satisfaction. The training also had a positive effect on stress reactions because the individual awareness (perception) of stress decreased significantly. Thus, it appears that the online training may be a useful tool in occupational health settings and among employees with high levels of work-related stress.

These results suggest that the training could be a tool with some additional health-promoting or medical relevance (e.g., for primary care, health promotion, and preventive settings). Hence, positive training effects were also seen in an increase in mindfulness, recovery experience, and flourishing. Moreover, effects of the training continued and were still significant, persisting 4 weeks after the end of the training (except for the muscular Stress Warning Signals and the variable of “flourishing” in the between-group analysis), thus pointing towards some sustained changes. The training may have initiated a process within the participants that had a long-term (at least 4 weeks) effect. However, underlying mechanisms and mediators of said effects still remain to be elucidated.

## Figures and Tables

**Table 1:** Short summary of the happiness trainings' exercises.

Week 1: basic principles	(i) How do you feel? Check your state of mind.
	(ii) What hindered you in the past from being happy?
	(iii) Write a happiness-diary! Note three things that made you happy today.

Week 2: joy of community	(i) Get some body's contact in a way that is comfortable for you.
	(ii) Identify your best friends and meet them this week.
	(iii) Write a thank-you letter.

Week 3: joy of luck	(i) Tell three people your wishes.
	(ii) Rejoice somebody by doing an unexpected favor.
	(iii) Let fortuity decide to do something new and give favorable opportunities a chance.

Week 4: joy of pleasure	(i) Eat a meal mindfully.
	(ii) Be mindful and capture happy moments with your camera.
	(iii) Challenge yourself with exercises/sports.

Week 5: joy of flow	(i) Identify your strengths.
	(ii) Use them in a new way.

Week 6: joy of bliss/beauty	(i) Give little presents to make somebody happy.
	(ii) Write a gratitude-diary and note three things a day you are thankful for.
	(iii) Enjoy ten minutes of silence every day.

Week 7: final	(i) Detect your favorite happiness exercises.
	(ii) Be a happiness messenger and tell your favorite exercises to other people.
	(iii) Reward yourself for your happiness-work during the last week and give yourself a treat.

**Table 2:** Study population of analyzed data.

	IG	CG
*N*	54	47
Female	41 (75.9%)	29 (61.7%)
Male	13 (24.1%)	18 (38.3%)
Age (in years)	37.61 (SD 7.715)	36.77 (SD 10.42)
Period of employment (in years)	15.89 (SD 8.536)	14.36 (SD 9.18)

**Table 3:** Descriptive statistics and between-group-effects of the questionnaire.

Questionnaire	*t*0					*t*1					*t*2				
*N* = 101	M	SD	Mann-Whitney *U *	*P*	*d*	M	SD	Mann-Whitney *U *	*P*	*d*	M	SD	Mann-Whitney *U *	*P*	*d*
Happiness															
IG CG	3.76 3.81	1.01 1.41	1164.5	0.461	0.04	4.52 3.38	1.13 1.34	603	0.000**	0.93	4.35 3.23	1.01 1.37	681	0.000**	0.92
Satisfaction															
IG CG	3.80 3.68	1.00 1.43	1265.5	0.980	0.10	4.54 3.19	1.13 1.19	532	0.000**	1.17	4.54 3.30	0.97 1.32	588.5	0.000**	1.10
Quality of life															
IG CG	13.09 12.04	4.17 5.61	1154	0.432	0.22	15.15 10.23	3.90 5.48	624	0.000**	1.06	15.04 10.55	4.32 5.34	664.5	0.000**	0.94
Stress Warning Signals-Sum															
IG CG	184.80 207.60	92.27 112.91	1157.5	0.448	0.22	147.28 212.32	87.57 117.32	839.5	0.003*	0.64	133.09 217.74	74.64 125.26	741.5	0.000**	0.84
Muscular															
IG CG	22.30 23.83	12.68 15.02	1234	0.812	0.11	18.91 25.43	11.94 14.43	930	0.021	0.50	18.52 23.30	11.61 14.96	1053.5	0.142	0.36
Vegetative- endocrinological															
IG CG	60.78 69.30	34.27 40.91	1109.5	0.277	0.23	50.22 71.17	29.54 43.99	900.5	0.012	0.57	46.19 73.72	27.22 47.52	808	0.002*	0.73
Cognitive															
IG CG	27.89 33.34	15.80 20.63	1133	0.354	0.30	22.59 33.15	16.23 19.51	878	0.008*	0.60	21.13 33.72	13.81 20.48	800.5	0.001*	0.74
Emotional															
IG CG	36.30 40.77	20.84 23.91	1141.5	0.385	0.20	26.93 42.00	20.65 23.87	757	0.000**	0.69	23.13 43.43	15.43 25.67	661.5	0.000**	0.98
Behavioral															
IG CG	37.54 40.36	22.08 26.48	1248	0.886	0.12	28.63 40.58	18.03 26.09	950.5	0.030	0.54	24.13 43.57	16.39 27.68	714	0.000**	0.88
Mindfulness															
IG CG	35.89 34.98	6.54 8.38	1139.5	0.377	0.12	39.43 35.17	6.42 7.43	866	0.006*	0.62	39.50 35.19	6.97 7.43	835	0.003*	0.61
Recovery experience															
IG CG	49.85 50.70	9.05 11.55	1266	0.770	0.08	54.57 47.81	9.47 12.29	824.5	0.002*	0.63	53.87 49.49	10.30 12.94	1006.5	0.074	0.38
Flourishing															
IG CG	42.74 43.04	6.51 8.62	1120	0.309	0.04	46.13 43.06	7.19 7.71	951	0.030	0.42	45.70 43.85	6.73 8.11	1104.5	0.262	0.25

*P < 0.01, **P < 0.001.

**Table 4:** Within-group effects (IG) of the questionnaire.

Within-group effects (IG)	t0-t1			t0–t2			t1-t2		
	P	Wilcoxon *Z *	d	P	Wilcoxon *Z *	d	P	Wilcoxon *Z *	d
Happiness	0.000**	−3.63	0.72	0.002*	−3.05	0.57	0.329	−0.98	0.16
Satisfaction	0.000**	−3.64	0.70	0.000**	−3.52	0.76	0.783	−0.28	0.00
Quality of life	0.004*	−2.88	0.51	0.012	−2.50	0.46	0.892	−0.14	0.03
Stress Warning Signals-Sum	0.012	−2.51	0.42	0.003*	−3.00	0.62	0.487	−0.70	0.18
Muscular	0.154	−1.43	0.28	0.095	−1.67	0.31	0.922	−0.10	0.03
Vegetative-endocrinol.	0.058	−1.90	0.33	0.022	−2.29	0.48	0.521	−0.64	0.14
Cognitive	0.019	−2.35	0.33	0.022	−2.29	0.46	0.856	−0.19	0.10
Emotional	0.003*	−2.95	0.46	0.000**	−3.58	0.87	0.410	−0.82	0.21
Behavioral	0.019	−2.35	0.45	0.001*	−3.47	0.70	0.205	−1.27	0.26
Mindfulness	0.010	−2.57	0.55	0.010	−2.58	0.54	0.873	−0.16	0.01
Recovery experience	0.019	−2.35	0.51	0.037	−2.09	0.42	0.718	−0.36	0.07
Flourishing	0.002*	−3.13	0.50	0.007*	−2.72	0.45	0.467	−0.73	0.06

*P < 0.01, **P < 0.001.

**Table 5:** Results of saliva samples.

Saliva *N* = 37	*t*0		*t*1		Between-group *t*1			Within-group (IG) *t*0-*t*1		
	M	SD	M	SD	Mann-Whitney *U*	*P*	*d*	Wilcoxon *Z*	*P*	*d*
*Alpha-Amylase (U/mL) *										
Awakening response										
IG CG	7.18 4.97	28.04 21.58	−12.41 17.77	55.37 47.22	112	0.073	0.60	−1.11	0.267	0.46
Morning activity										
IG CG	35.67 33.35	22.79 19.56	59.95 78.74	36.03 117.96	161	0.761	0.22	−3.11	0.002*	0.83
Evening activity										
IG CG	109.87 121.62	90.46 82.88	114.45 126.89	92.94 74.24	145	0.429	0.15	−0.63	0.528	0.05
Circadian rhythm amplitude										
IG CG	−74.30 −88.27	81.15 83.36	−54.49 −48.16	79.48 109.03	160	0.738	0.07	−1.20	0.231	0.25
Raw data										
Directly after awakening										
IG CG	32.08 30.86	20.55 20.12	66.16 69.85	47.41 107.72						
30 minutes after awakening										
IG CG	39.27 35.83	31.78 24.36	53.75 87.62	43.38 131.68						
At 8 pm										
IG CG	109.87 121.62	90.46 82.88	114.45 126.89	9294 74.24						
*Cortisol (nmol/L) *										
Awakening response										
IG CG	7.48 4.32	10.68 11.23	6.82 4.47	11.59 13.27	126	0.171	0.19	−0.24	0.811	0.06
Morning activity										
IG CG	31.47 30.95	9.65 15.60	29.37 31.05	10.01 11.19	157.5	0.682	0.16	−0.85	0.396	0.22
Evening activity										
IG CG	2.67 3.51	1.84 3.82	5.95 4.56	13.11 7.59	147	0.466	0.13	−0.00	1.000	0.36
Circadian rhythm amplitude										
IG CG	28.79 27.43	9.60 15.77	23.42 26.49	16.43 13.30	155	0.627	0.21	−1.11	0.267	0.41
Raw data										
Directly after awakening										
IG CG	27.73 28.79	7.79 16.54	25.96 28.82	11.68 13.19						
30 minutes after awakening										
IG CG	35.21 33.11	13.52 16.62	32.78 33.29	11.45 12.83						
At 8 pm										
IG CG	2.67 3.51	1.84 3.82	5.95 4.56	13.11 7.59						

*P < 0.01, **P < 0.001.

**Table 6:** Results of Attention Network Test (ANT).

	*t*0		Between-group		*t*1		Within-group			
ANT N = 27							*t*0-*t*1 IG		*t*0-*t*1 CG	
	M	SD	Mann-Whitney *U*	P	M	SD	Wilcoxon *Z*	P	Wilcoxon *Z*	P
Alertness										
IG CG	39.2 35.2	17. 0 24.2	81	0.730	50.9 48.6	22.1 18.9	1.58	0.115	1.56	0.119
Orienting										
IG CG	51.4 56.5	29.4 30.9	100	0.554	39.5 37.7	21.5 21.3	−2.77	0.006	−1.51	0.130
Conflict										
IG CG	140.7 186.0	40.2 102.8	106	0.374	119.3 128.5	22.9 58.7	−2.41	0.016	−2.85	0.004
Reaction time										
IG CG	658.7 671.0	65.3 71.4	95	0.711	641.3 619.0	58.8 55.5	−1.82	0.069	−2.94	0.003
Accuracy										
IG CG	95.9 96.9	2.3 2.0	109	0.293	97.4 98.9	1.6 0.7	2.65	0.008	2.7	0.007

**Table 7:** Most significant correlation coefficients of IG (differences Δ between *t*1-*t*0).

Correlations IG	Quality of life	Happiness	Satisfaction	Mindfulness	Alpha-Amylase Morning Activity
Δt1 − t0	*r*	*r*	*r*	*r*	*r*
Happiness	0.375**		0.575**	0.301*	
Satisfaction	0.400**	0.575**	—	0.445**	0.850**
SWS—emotional	−0.446**	−0.476**	−0.454**	−0.346*	
SWS—sum	−0.454**	−0.517**	−0.484**	−0.330*	
Flourishing	0.339*	0.397**	0.424**	0.600**	

*P < 0.05, **P < 0.01.

## Abbreviations

IG =: Intervention group
CG =: Control group
*P* =: Significance, (**P* < 0.01, ***P* < 0.001)
*d* =: Cohen's *d*
*r* =: Correlation coefficient
*M* =: Mean
SD =: Standard deviation.
